# Electrical Impedance Myography to Detect the Effects of Electrical Muscle Stimulation in Wild Type and *Mdx* Mice

**DOI:** 10.1371/journal.pone.0151415

**Published:** 2016-03-17

**Authors:** Jia Li, Sung Yim, Adam Pacheck, Benjamin Sanchez, Seward B. Rutkove

**Affiliations:** From the Department of Neurology, Beth Israel Deaconess Medical Center, Boston, Massachusetts, 02215, United States of America; University of Sydney, AUSTRALIA

## Abstract

**Objective:**

Tools to better evaluate the impact of therapy on nerve and muscle disease are needed. Electrical impedance myography (EIM) is sensitive to neuromuscular disease progression as well as to therapeutic interventions including myostatin inhibition and antisense oligonucleotide-based treatments. Whether the technique identifies the impact of electrical muscle stimulation (EMS) is unknown.

**Methods:**

Ten wild-type (*wt*) C57B6 mice and 10 dystrophin-deficient (*mdx*) mice underwent 2 weeks of 20 min/day EMS on left gastrocnemius and sham stimulation on the right gastrocnemius. Multifrequency EIM data and limb girth were obtained before and at the conclusion of the protocol. Muscle weight, *in situ* force measurements, and muscle fiber histology were also assessed at the conclusion of the study.

**Results:**

At the time of sacrifice, muscle weight was greater on the EMS-treated side than on the sham-stimulated side (p = 0.018 for *wt* and p = 0.007 for *mdx*). Similarly, in *wt* animals, EIM parameters changed significantly compared to baseline (resistance (p = 0.009), reactance (p = 0.0003) and phase (p = 0.002); these changes were due in part to reductions in the EIM values on the EMS-treated side and elevations on the sham-simulated side. *Mdx* animals showed analogous but non-significant changes (p = 0.083, p = 0.064, and p = 0.57 for resistance, reactance and phase, respectively). Maximal isometric force trended higher on the stimulated side in *wt* animals only (p = 0.06). Myofiber sizes in *wt* animals were also larger on the stimulated side than on the sham-stimulated side (p = 0.034); no significant difference was found in the *mdx* mice (p = 0.79).

**Conclusion:**

EIM is sensitive to stimulation-induced muscle alterations in *wt* animals; similar trends are also present in *mdx* mice. The mechanisms by which these EIM changes develop, however, remains uncertain. Possible explanations include longer-term trophic effects and shorter-term osmotic effects.

## Introduction

Electrical muscle stimulation (EMS) to improve muscle condition and function in both health and disease has been studied since the beginning of the 20^th^ century [1]. While the method has been applied to a wide variety of disorders, its actual therapeutic value in certain conditions has been questioned. For example, a 1943 article studying the effects of EMS in denervated muscles in humans argued that it was ineffective [2]. However, in non-denervated muscle tissue—namely that impacted by chronic disuse, primary muscle disease, including muscular dystrophy, and central disorders—EMS continues to be actively studied and used. For example, studies have shown some modest but clinically meaningful effects in boys with Duchenne muscular dystrophy [3–5]. Other more recent work has demonstrated improvement in muscle condition in patients in the intensive care unit [6], with spinal cord injury [7], and in cerebral palsy [8]. More recently, EMS has also been shown to serve as a potential therapy for sarcopenia [9]. Finally, EMS supplements standard exercise training in sports [10–12].

Approaches for evaluating the effect of EMS on muscle condition vary and include relatively simple metrics such as measuring function [10–12] and, in animal studies, excised muscle weight. Magnetic resonance imaging [13] and ultrasound [14] have also been applied. However, one technique that may offer a simple non-invasive approach to the evaluation of muscle condition and the effects of EMS is electrical impedance myography (EIM). Since EIM is easy to employ and is sensitive to muscle fiber size and muscle volume [15], its application specifically for the assessment of EMS efficacy could be of wide value to the medical and sports communities. As an initial exploration of this idea, here we study groups of wild type (*wt*) and dystrophin-deficient (*mdx)* animals whose hind limbs were treated unilaterally with EMS for two weeks.

## Methods

### Mice

**Fig 1:**
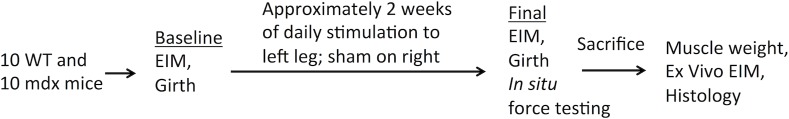
Schematic of study design.

All studies were approved by the Beth Israel Deaconess Medical Center Institutional Animal Care and Use Committee. Ten male wild-type (C57BL/6J) and 10 male *mdx* (C57BL/10ScSn-Dmd^mdx^/J) mice were obtained from Jackson Laboratories (Bar Harbor, ME) and were 9–10 weeks of age at study commencement. An outline of the study design is shown schematically in Fig 1.

### Animal measurement preparation

Baseline animal measurements were performed under 1–2% inhaled isoflurane anesthesia delivered by nosecone, after whole body weight was obtained, with body and muscle temperature being maintained by a heating pad (37°C). The fur on both hind limbs was clipped and a depilatory agent was applied to the skin to remove all remaining fur. Then the skin was then cleaned with 0.9% saline solution. The leg was taped to the measuring surface at an approximately 45° angle extending out from the body. This set up was used both for EIM measurements and for daily application of EMS, as described below.

### Baseline measurements

#### Limb girth

Limb girth was measured by measuring the widest area of the distal hind limb (calf-shin) with a thread. Measurements were made 3 times and averaged.

#### EIM measurements

An impedance analyzer (EIM1103, Skulpt Inc., San Francisco, California, USA) was used to obtain multifrequency data (at 40 discrete frequencies) between 1 kHz and 10 MHz. The EIM 1103 system is built around a low-input-impedance, wide-bandwidth, transimpedance amplifier and low-capacitance, high-bandwidth junction gate field-effect transistor differential amplifier to minimize parasitic capacitance. The EIM 1103 uses high-speed analog-to-digital converters to measure amplified signals directly, which then incorporate data from a lock-in amplifier digitally. The phase and magnitude errors from second-stage amplifiers and anti-aliasing filters are minimized by simultaneously measuring voltage and current signals using two matched channels. Bench testing of the EIM 1103 system confirms a highly accurate impedance-frequency curve compared to other commercially available devices and compared to SPICE (Simulation Program with Integrated Circuit Emphasis) simulations, with offsets of below 1% from 1 kHz to 1 MHz as compared to theorized performance. Test-retest reliability is within 5–10% for both healthy subjects [16] and mice [17]; intraclass correlation values are also over 0.90 [16,17].

An electrode array made of four parallel stainless steel strips embedded into a plastic block was used to perform the measurements as previously described [18].

### Electrical muscle stimulation protocol

The left distal hind limb (encompassing both the anterior and posterior compartments) underwent electrical stimulation with a commercially available electrical stimulator for sports use—the Compex Sport Elite Muscle Stimulator (Surrey, United Kingdom) for 20 minutes. Stimulation was provided by a pair of ring electrodes placed around the distal limb (Catalogue # 9013S0312, Natus Neurology, Middleton, Wisconsin, USA).

We used the muscle endurance program set to level 1 with intensity 40 (available range from 1–999). While the manufacturer does not provide the details of the stimulation protocol, we utilized an oscilloscope to further characterize it. We found the pulse was a biphasic square wave with a width of about 800 microseconds. The intense part of the stimulation delivered the pulse at a frequency of 10 Hz and a current of 50 mA for 9 seconds. The recovery portion lasted 2 seconds with pulses at a frequency of 3 Hz and a current of approximately 33 mA. The stimulation repeated every 11-seconds.

The right side underwent analogous sham stimulation. Specifically, electrodes were placed around the distal limb but no electrical stimulation was applied. EMS and sham therapy continued for approximately 12–14 days on each animal (there was small variation as to the exact number of days depending upon when a given animal underwent the final testing, since they were performed over a several day period). All animals not undergoing final assessments, including *in situ* force measurements, continued to receive stimulation up until the day before sacrifice.

### Measurements at study conclusion

Limb girth measurements and EIM data collection were repeated.

#### *In situ* force measurement

This procedure is described fully elsewhere [19]. Immediately after the above testing was completed, the gastrocnemius was surgically exposed and the calcaneal tendon was then cut at its insertion point and dissected away from the underlying fascia and soleus muscle. The tendon was then connected to a force lever arm of a high-speed servomotor-based apparatus (Model 305C, Aurora Scientific, Aurora, Ontario, Canada). The leg was stabilized by inserting a disposable monopolar needle (902-DMF37-S, Natus neurology, Middleton, Wisconsin, USA) through the knee joint. Twitch force was recorded with a 200 *μ*s square pulse stimulated at different rates delivered to insulated electrocardiogram needles (F-E2M-48, Grass Technologies, Warwick, Rhode Island, USA) stimulating the sciatic nerve at the sciatic notch via a current muscle stimulator (Model 701, Aurora Scientific). The output force-length signals from the lever system were interfaced to a PC-platform based on a PXI system integrating a PXIe-8135 quad-core processor based embedded controller and a two-channel acquisition board PXI-4461 (National Instruments, Austin, Texas, USA).

Stimulation current and muscle resting tension were adjusted to maximize tetanic force. Optimal current and resting tension were determined by maximizing the force produced by a single stimulus pulse. All subsequent isometric data were collected at this stimulation current and resting tension. Tetanic force frequency/relationship was recorded after stimulation by a train of square wave stimuli of 200 ms.

Muscle weight: Immediately after completion of the *in situ* measurements above, the animals were sacrificed and the gastrocnemius muscles were removed and weighed with a standard analytical balance.

Histological assessment: Excised gastrocnemius muscle tissue was flash frozen in isopentane, which had been cooled by liquid nitrogen to -80°C. Tissue corresponding to the largest part of the muscle was cut into 5 μm slices, placed on glass slides, and stained with hematoxylin and eosin. Utilizing a computer (Dell Optixflex 380) and Zeiss Axiophot microscope with a motorized stage, counting a total of approximately 60 fibers per muscle. An evaluator quantified myofiber size using Stereo Investigator software (MBF Biosciences Inc., Williston, Vermont, USA) and was blinded from treatment and disease status. Myofiber area was estimated by manually delimiting each single myofiber contour and approximating the shape by an octagon.

### Data Analysis

All EMS-treated to sham-stimulated limb comparisons for both groups of animals were performed using paired t-tests. For EIM multifrequency data, only values from 10 to 1000 kHz are presented. The reason for this limited range is that below 10 kHz, inconsistency in the skin-electrode contact interface creates artifacts in the acquired data; over 1000 kHz, the inductive properties inherent to the measurement set-up also create major distortions in the data. Whereas there are methods for experimentally correcting for these high-frequency inductive artifacts (see Sanchez et al [20]), such efforts are complex and well beyond the scope of this study. In addition, muscle is most reactive in the 30–200 kHz range [21]. Accordingly, for our single frequency analyses, we analyzed the 50 kHz data, in keeping with much of our own previous work.

Data analysis of the *in situ* force measurement required a modified approach. The reason for this is that the animals were required to be under anesthesia for an extended period of time before both of the surgeries could be completed. We quickly identified that the muscle being studied second consistently demonstrated very low force generation, likely due to prolonged anesthesia [22]. Accordingly, we intentionally studied the sham treated leg in half the animals first and the EMS-treated leg in the other half. This allowed us to perform a fair comparison of force generating abilities of EMS treated versus sham stimulation via application of unpaired t-tests, although this decreased N to 5 for each comparison.

Significance for all paired and unpaired t-tests was set at alpha = 0.05, two-tailed. Data reported represents the mean ± standard error of the mean (SEM).

## Results

Detailed results are provided in S1 Table.

### Girth and muscle weight

**Fig 2:**
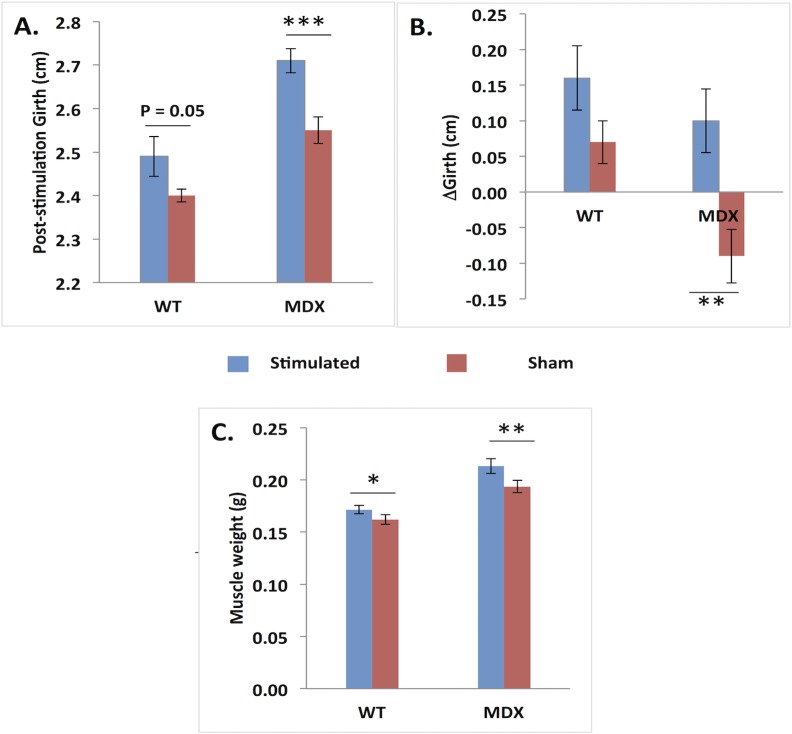
Post-stimulation/sham-stimulation and change in limb girth (a and b) and post-stimulation differences for muscle weight (c). *p < 0.05, **p < 0.01, ***p< 0.001.

Fig 2 shows limb girth at baseline and at the time of sacrifice and muscle weight at the time of sacrifice. There is significantly greater muscle weight in the EMS-treated versus the sham-stimulated limbs for both *mdx* and *wt* animals (p = 0.018 and p = 0.007, respectively). Girth differences are most apparent in the *mdx* mice and are only borderline significant in the *wt* animals.

### EIM data

**Fig 3:**
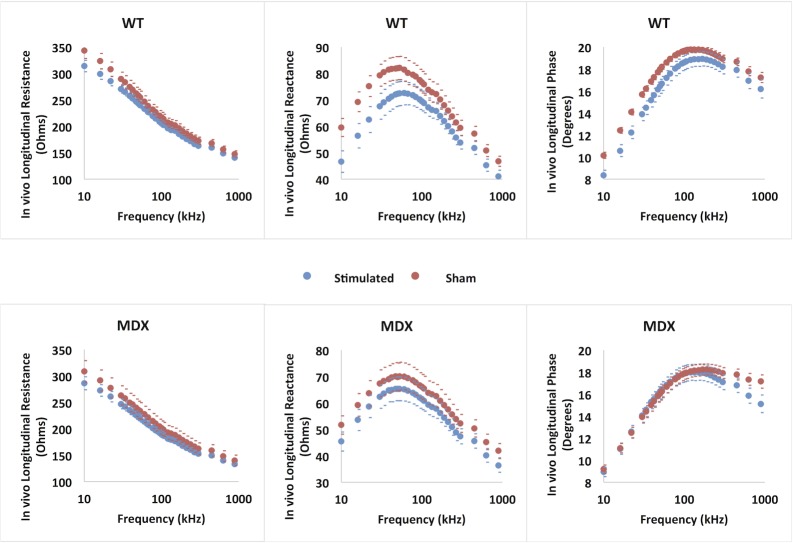
Post-stimulation/sham-stimulation multifrequency (+/- SEM) data for the 3 major impedance parameters, reactance, resistance, and phase, for both *wt* animals (a-c) and *mdx* animals (d-f). Note that the y-axis scales have each been tailored to highlight the differences in impedance values between the two limbs.

**Fig 4:**
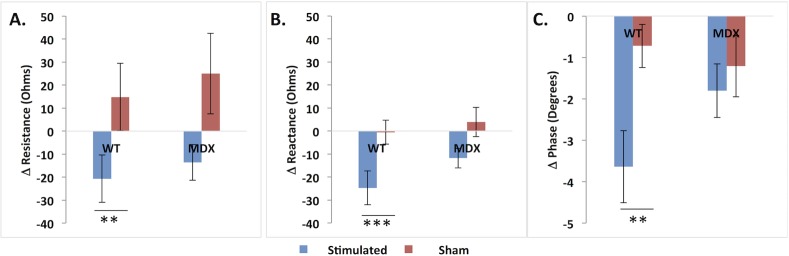
Pre-post changes in 50 kHz impedance parameters for stimulated and sham-stimulated limbs. Note reductions in all 3 parameters with stimulation. **p < 0.01, ***p < 0.001).

Fig 3 shows the mean (±SEM) multifrequency EIM resistance, reactance and phase data for both the *wt* and *mdx* animals comparing stimulated and sham-stimulated limbs at sacrifice. The differences for the *mdx* animals are smaller than those for the *wt* animals across the frequency spectrum. Fig 4 shows the corresponding changes (baseline-final measurement) in 50 kHz values. There are consistent changes in both *mdx* and *wt* animals; the changes in the *wt* animals reach significance (p = 0.009 for resistance, 0.0003 for phase, and 0.002 for resistance), whereas *mdx* changes do not (p = 0.083 for resistance, 0.064 for phase, and 0.57 for phase).

### *In-situ* force measurements

**Fig 5:**
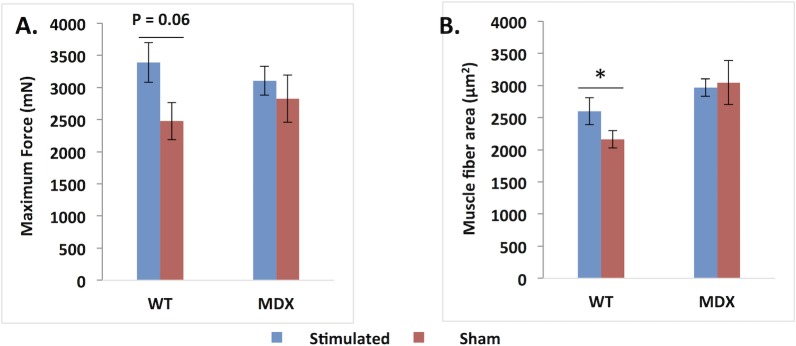
A. *In situ* maximal isometric force with tetanic stimulation in *mdx* and *wt* mice post-stimulation/sham-stimulation. For these analyses, only the side that underwent stimulation first was utilized in the analysis, thus N = 5 for each group in each of these unpaired comparisons. B. Stimulated versus sham-stimulated muscle fiber size in both *wt* and *mdx* mice (N = 9 and 8, respectively). *p < 0.01.

We compared the sham-stimulated legs to the stimulated legs for *mdx* and *wt* animals. As Fig 5A shows, in general there is a relatively greater force in the EMS-treated side as compared to the sham-stimulated side, although this effect is much more apparent for the *wt* than for the *mdx* mice, reaching near-significance (p = 0.06) in the *wt* animals.

### Histological assessments

Fig 5B shows a significant elevation in muscle fiber size (p = 0.034) in the EMS-treated limb of the *wt* animals as compared to the sham-stimulated limb. In the *mdx* mice, the greater muscle fiber size in the stimulated limb was non-significant.

## Discussion

The girth, muscle weight, EIM, and force data support the hypothesis that direct electrical stimulation of muscle fibers for short intervals (i.e., 20 minutes per day) and a relatively short period of time (2 weeks) can produce significant alterations in muscle. Our histologic assessment suggests that in the *wt* animals, part of this hypertrophy was due to increased myofiber size, resulting in greater force production, and significantly different EIM values. However, the *mdx* animals showed non-significant changes in myofiber size and force production and correspondingly weaker differences in EIM values. Whereas the concept of using EMS to improve muscle condition is not new, to our knowledge, this is the first study to pursue the question in *mdx* mice; previous work in *mdx* mice used electrical stimulation of the sciatic nerve rather than direct stimulation of the muscle [23–25].

Why the *mdx* mice demonstrated no significant muscle fiber hypertrophy or increase in strength despite the increase in girth and muscle weight could be due to several factors. First, the addition or subtle enlargement of dystrophic myofibers may not be reflected in improved force outcome. Since previous studies have demonstrated a beneficial impact of sciatic nerve stimulation on strength in *mdx* mice [23–25], perhaps a stronger stimulation paradigm for a longer period of time would have produced more substantive changes in muscle fiber size and force. A second explanation is that we induced simultaneous trophic and injurious effects in the *mdx* mice. *Mdx* muscle is more prone to exercise induced injury [26]; thus the same stimulation paradigm could induce hypertrophy in only *wt* animals and simultaneous hypertrophy and injury in *mdx* animals, and thus counteracting any anticipated improvements in force.

We hypothesized that EIM data should be sensitive to alterations in muscle induced by EMS. And, indeed, such changes were observed. However, the direction of the anticipated change was unexpected. Specifically, we had predicted that increasing muscle fiber size would lead to elevations in the reactance and phase with less substantial effects on the resistance. The reason we anticipated an increase in reactance (and subsequently in phase) is that enlarged muscle membrane area within the tissue should increase the momentary charge storage capability of the tissue. To our surprise, the results showed substantial reductions compared to baseline in all three parameters in the *wt* animals. This result is even more perplexing in the face of the observed significant increase in muscle fiber size in the *wt* animals.

There are two major factors that may be contributing to this unexpected result and may also help explain why the *mdx* muscle showed increased weight and girth but no significant change in EIM parameters. The first is the effect of overall muscle volume. If we were to artificially enlarge the muscle volume by adding myofibers (and not change the intrinsic structure of the fibers themselves), the greater volume would cause the current density to decrease and thus reduce the measured impedance parameters. We have previously observed this effect in rats, in which we witnessed reductions in reactance and resistance as young animals matured, despite their having larger myofiber cross sectional area [27]. More recently, we have modeled this effect in the human tongue and similarly observed that increasing muscle size alone, keeping all other parameters identical, causes reductions in the measured resistance and reactance [28]. This also is due to a lower current density in the region of the voltage electrodes.

For this explanation to be accurate, it would require that there be a concomitant increase in myofiber number, along with the identified myofiber hypertrophy. In fact, one of the earliest studies of EMS in *mdx* mice [23] actually demonstrated an increase in muscle fiber number with sciatic nerve stimulation. Other studies have also demonstrated activation of satellite cells, further supporting the idea that EMS can lead to increases in myofiber number [29–31]. Some of these studies were done in the setting of induced disuse atrophy, so these results do have to be applied to the current study with some caution. Nonetheless, it is possible that increased fiber number caused an increase in muscle volume, counteracting the effects of the myofiber hypertrophy on the impedance values. Unfortunately, we did not measure satellite cell activation or count the actual number of myofibers in the muscle.

The second explanation moves away from the purely trophic effects of exercise on muscle, whether it is increasing myofiber number or myofiber hypertrophy, and invokes the possibility of short-term osmotic shifts due to EMS-induced muscle contraction. Magnetic resonance imaging studies have shown that exercise causes muscle fiber swelling and increased extracellular water [32–34]. These changes have also been observed in muscle after EMS [35]. Such osmotic alterations might also be expected to change the tissue’s electrical properties. It may also be for this reason that the *mdx* muscle shows a substantial change in girth without a clear change in muscle fiber size or increased strength. This may be especially true in this situation because we continued to perform EMS until the day prior to the final EIM data collection in all animals. Of course, it is also possible that a combination of factors is at work—both long-term trophic effects causing greater muscle volume and short-term osmotic alterations further increasing the intra- and extracellular water in the muscle.

This discussion ignores one other important issue that adds further complexity to the interpretation of the data: namely that the sham-stimulated side showed substantial changes as well. These changes included a reduction in girth in the *mdx* animals and elevations in resistance and to a lesser extent reactance, also most clearly in the *mdx* animals. Several studies have previously identified contralateral effects with unilateral exercise or EMS [36–40]. In these cases, the non-stimulated contralateral side showed subtle muscle hypertrophy. Moreover, one recent study confirmed that overuse and exercise-induced injury can also be “transmitted” to the contralateral side [41]. In our study, the sham-stimulated side actually showed changes opposite to those of the stimulated side. Thus, the only explanation that we can offer is that the animals were using the EMS-treated limb preferentially during daily activity, resulting in some subtle disuse change on the sham-stimulated side.

To better understand what mechanisms are at play in causing alterations on both EMS-treated and sham-stimulated sides, we would need to modify the study with varying time delays between the final stimulation period and data collection. Adding a separate second modality (e.g., magnetic resonance imaging) may also provide valuable information. Another possibility would be to perform studies on larger numbers of mice and sacrificing animals at multiple time points to understand more clearly the effects of stimulation on EIM and muscle pathology, both on the stimulated and sham-stimulated sides.

There are a variety of additional limitations to this study that are worth highlighting. First, we arbitrarily chose a stimulation paradigm that was based solely on our observation that the muscle appeared to be contracting vigorously. Previous studies have sought to identify optimal stimulation protocols [30]; however, since we were using a commercial device with preset programs, it was not possible to fine-tune our protocols. Second, we did not perform any cellular assays to evaluate satellite cell activation or other measures to determine if there were an increase in the total number of myofibers present. Third, we were unable to perform side-to-side comparisons on our *in situ* force measurements, limiting that analysis. Finally, we do not provide a detailed review of the multifrequency EIM data analysis here, outside of presenting the plots in Fig 2. However, subsequent analyses (not shown) failed to reveal any additional findings of interest beyond those demonstrated at 50 kHz.

In summary, these results show that EMS can produce significant changes in EIM data over just a 2-week period in *wt* animals, with analogous although less dramatic changes in *mdx* animals. Given the unexpected direction of EIM alterations, to better understand the mechanism underlying these findings, further study of this effect is clearly needed. But mechanisms aside, the fact that EIM can detect EMS-induced muscle alterations supports the basic premise that the technique has the potential to monitor the impact of EMS on muscle condition in both health and disease.

## Supporting Information

S1 Table Data values obtained for function measures, weight, girth and electrical impedance myography are provided. Stim, simulated limb data; sham, sham stimulated limb data; mdx, muscular dystrophy; wt, wild-type.
(XLSX)
